# Limitations of ITA for Skin Type Estimation Under Uncontrolled Imaging Conditions

**DOI:** 10.1111/srt.70374

**Published:** 2026-08-11

**Authors:** Neda Alipour, Ted Burke, Jane Courtney

**Affiliations:** ^1^ School of Electrical and Electronic Engineering Technological University Dublin Dublin Ireland

**Keywords:** Fitzpatrick skin type, ITA, lighting variation, skin type measurement

## Abstract

**Background:**

Accurately assessing skin color diversity is essential for evaluating whether image datasets without explicit skin type labels are sufficiently diverse for training deep learning models. Traditional methods, such as the Fitzpatrick scale, categorize skin types into six discrete classes but may not fully capture the complexity of skin color and its interaction with light under varying conditions. Continuous and quantitative approaches have been proposed to better represent skin color variation, but their reliability under uncontrolled imaging conditions remains unclear.

**Materials and Methods:**

This study evaluated individual typology angle (ITA), an image‐derived color or lightness metric commonly mapped to Fitzpatrick skin type (FST) categories. A dermatologist‐labeled dataset (PAD‐UFES‐20) was used to analyze the performance of ITA. A skin patch image dataset was derived from PAD‐UFES‐20, and skin color features were extracted for evaluation.

**Results:**

The results showed substantial overlap between ITA distributions across FST categories and poor agreement with dermatologist‐assigned labels. Fixed‐threshold ITA classification achieved 22.9% accuracy, below the majority‐class baseline accuracy of 51.3%, and failed to correctly classify the darkest skin type (FST VI). Lighting variation, threshold misalignment, overlapping ITA distributions, and dataset imbalance contributed to unstable ITA behavior across skin types.

**Conclusion:**

Under uncontrolled imaging conditions, fixed‐threshold ITA did not provide reliable Fitzpatrick skin type classification and should not be interpreted as a validated skin type measurement method. These findings demonstrate that ITA is highly sensitive to lighting variation and does not reliably correspond to dermatologist‐assigned FST labels. The results highlight the limitations of using ITA as a proxy for skin type assessment in image datasets.

## 1 Introduction

**FIGURE 1:**
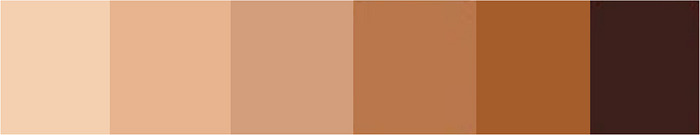
The FST system categorizes various skin colors from type I (very light) to type VI (dark).

Ensuring skin type diversity is essential for AI‐based skin analysis applications, as it affects the accuracy of tasks such as detecting skin conditions and evaluating treatment outcomes [1, 2]. Studies like Gender Shades have highlighted how disparities in dataset representation can lead to significant performance gaps in AI systems, particularly for underrepresented groups such as individuals with darker skin types [3]. Reliable measurement of skin type diversity is crucial for creating datasets that allow AI models to generalize across different skin types. In dermatology, the Fitzpatrick skin type (FST) is the most used system to classify skin type, providing a standard for healthcare professionals and aesthetic practitioners [4]. This system involves a face‐to‐face meeting with dermatologists [5, 6]. The Fitzpatrick scale, which categorizes skin based on its color and reaction to initial sun exposure resulting in erythema (burning or tanning), was originally developed by Thomas B. Fitzpatrick in 1975 [7]. This classification system is subjective, as it relies on self‐reporting or dermatologist assessment rather than quantitative metrics. These six skin types are shown in Figure 1.

An alternative extension to the Fitzpatrick classification is the Monk Skin Tone (MST) [8] scale, a 10‐point system developed by Ellis Monk to more comprehensively represent the diversity of human skin tones in fairness‐focused AI applications. However, like Fitzpatrick, it discretizes the continuous skin color spectrum into fixed categories and therefore cannot fully capture the nuanced variations observed in real‐world skin colors.

Prior research has shown that using automated methods, skin type assessment can be done directly from images [9, 10]. However, there are some limitations when detecting exact skin types using automatic methods; for instance, the methods face challenges in determining skin type in images taken under different lighting conditions and may not perform well for all skin type groups. Therefore, a comprehensive review of existing skin type measurement methods used in previous studies was conducted to identify which methods were most frequently used and whether the limitations were considered for each method.

An overview of studies that have used different skin type measurement methods is presented in Table 1. For example, the importance of accounting for varying lighting conditions in skin type measurements lies in its direct impact on skin color perception, texture representation, and the overall quality of captured images. Fitzpatrick labels can also give insight into the different skin types utilized to develop these methods. Table 1 shows a comparative overview of skin type classification and measurement methods used in peer‐reviewed publications. Papers were selected as follows: A systematic search was performed on the PubMed, IEEE Xplore, and Google Scholar databases using the search terms “Fitzpatrick skin type”, “illumination variation”, and “skin type measurement”. The comparison included only peer‐reviewed journal papers and peer‐reviewed conference papers from those search results. The investigation revealed that various methods are used to measure skin type, ranging from physical devices like spectrophotometers to computational approaches, including AI‐based methods, ITA (individual typology angle), and others.

**TABLE 1:** A comparative overview of skin type classification methods includes light variation consideration and FST label inclusion. Among these methods, individual typology angle (ITA) has been the most widely used in previous studies.

Reference	Year	Method	Light variation	Dermatologist‐assigned FST label	Image dataset with FST label
Meinhardt et al. [11]	2008	Minimal Erythema Dose (MED)/ ITA	No	No	−
Girardeau et al. [12]	2009	ITA	No	No	−
Reeder et al. [13]	2010	ITA	No	No	−
Huixia et al. [14]	2012	ITA	No	No	−
Arce‐Lopera [15]	2012	ITA	No	No	−
Bino & Bernerd [16]	2013	ITA	No	No	−
Huang et al. [17]	2013	Haar transform & Fuzzy	No	No	−
Battie [18]	2014	ITA	No	No	−
Reeder [19]	2014	ITA	No	No	−
Treesirichod et al. [20]	2014	Spectrophotometer	No	No	−
Ryu et al. [21]	2015	Spectrophotometer / ITA	No	No	−
Wilkes et al. [22]	2015	ITA	No	No	−
Youn [23]	2015	Sebumeter	No	No	−
Ash et al. [24]	2015	Skin Tone Meter (STM)	Yes	No	−
Cinotti et al. [25]	2016	ITA	No	No	−
McCreath et al. [26]	2016	Munsell color chart	Yes	No	−
Piazena et al. [27]	2017	Spectrophotometer / ITA	No	No	−
Choi & Suk [28]	2017	Spectrophotometer / ITA	No	No	−
Wang et al. [29]	2018	ITA	No	No	−
Chang et al. [30]	2018	AI	No	No	−
Kinyanjui et al. [31]	2019	ITA	No	No	−
Wang et al. [32]	2019	ITA	No	No	−
Campiche et al. [33]	2019	ITA & IWA_Newtone_	No	No	−
Humber et al. [34]	2019	DWT & LBP	No	No	−
Kinyanjui et al. [35]	2020	ITA	No	No	−
Indriyani & Sudarma [36]	2020	SVM	No	No	−
Ly et al. [37]	2020	Spectrophotometer / ITA	Yes	No	−
Bahmani et al. [38]	2021	SREDS	Yes	No	−
Krishnapriya et al. [39]	2021	ITA	Yes	No	−
Kothari et al. [40]	2021	AI	No	No	−
Flament et al. [41].	2021	AI	No	No	−
Tadesse et al. [42]	2021	ITA	No	No	−
John et al. [43]	2021	FALMS	No	No	−
Bevan & Abarghouei [44]	2022	ITA	No	No	−
Feng et al. [45]	2022	ITA	Yes	No	−
Groh et al. [46]	2022	ITA	No	Yes	Fitzpatrick 17k
Li et al. [47]	2022	ITA	No	No	−
W et al. [48]	2022	AI	No	No	−
Jung et al. [49]	2023	ITA	No	No	−
Kalb et al. [50]	2023	ITA	Yes	No	−
Barrett et al. [51]	2023	ITA	No	No	−
Corbin & Marques [52]	2023	ITA	No	No	−
Mohamed et al. [53]	2023	ITA	No	No	−
Thong et al. [54]	2023	ITA & Hue angle	No	No	−
Tadesse et al. [55]	2023	AI	No	Yes	Fitzpatrick 17k
Leizaola et al. [56].	2023	AI	No	No	−
Kara et al. [57]	2023	AI	No	No	−
Oliveira et al. [58]	2023	Human annotator	No	No	−
Schumann et al. [59]	2024	Human annotator	Yes	No	−
Srimaharaj & Chaising [60]	2024	Distance‐based	No	No	−
Mbatha et al. [61].	2024	AI	Yes	No	−
Draelos et al. [62].	2025	AI	No	No	−
Dissanayake & Manukalpa [63]	2025	AI	No	No	−
Paxton et al. [64].	2026	AI	No	No	−

As Table 1 shows, ITA is the dominant skin type measurement method in existing literature. This method measures skin pigmentation in the L^*^a^*^b^*^ color space, while the discrete and subjective Fitzpatrick categories are based on the pigmentation and response to UV exposure. Although ITA provides a continuous image‐derived representation of skin color, it can also be mapped onto discrete categories, like Fitzpatrick classification system. Some previous research has treated ITA classifications as synonymous with those of Fitzpatrick [65, 66]. ITA is not a classification method itself, but it is a feature used in skin type assessment, representing the angle between the color and luminance vectors of a person's skin color in the L^*^a^*^b^*^ color space, to estimate image‐derived skin color characteristics based on the calculated angle.

However, other existing skin type measurement methods, as outlined in Table 1, are physically based techniques, which require in‐person assessment and are therefore not suitable for evaluation through digital images. For example, skin reflectance estimation based on dichromatic separation (SREDS) was proposed to measure skin color from a single image by decomposing the light reflected from the skin into two components [38]. Three datasets, the CMU Multi‐PIE face database, MEDS‐II, and Morph‐II—were used to evaluate the superior performance of the new model compared to ITA and Relative Skin Reflectance (RSR). Although this method outperforms other methods in uncontrolled lighting conditions, it still has difficulties with variations in lighting conditions. In [43], face area lightness measures (FALMs) are calculated to estimate skin color from images using color analysis or FSTs. The estimated FALMs from the images are then compared to ground truth skin measurements from a calibrated dermatological device specifically designed to measure skin lightness, showing a large difference between these two measures resulting from camera, background, and environmental conditions.

In the case of deep learning‐based methods, which typically require large, diverse datasets and often do not use reliable or consistent Fitzpatrick labels. In many prior works, dermatologist‐assigned FST labels were either unavailable or generated using non‐expert annotators, which undermines the reliability of comparisons. Chang et al. [30] introduce a convolutional neural network model to classify their created skin spectra dataset into the six FSTs, assessing sun exposure reactions. Their performance evaluation includes a comparison with an artificial neural network model. Mbatha et al. [61]. developed a neural network model trained on human‐annotated data under 15 different lighting conditions to predict MST values with improved robustness. Their work demonstrates how modern machine learning techniques can produce consistent estimates across variable environments. However, such approaches typically require large, well‐labeled datasets and careful control of image acquisition, which may not always be available in real‐world or legacy image collections. The MST labels used for training and evaluation were manually assigned by the participants themselves. The classification of facial features extracted from images into different skin types of normal, oily, dry, and combination is accomplished using a support vector machine with the use of discrete wavelet transform, contrast, and local binary pattern [34]. A multi‐task cascaded convolutional neural network is used to classify skin images into skin types, oily or dry [40]. In another study, a biological approach is adopted, focusing on the detection of skin type through the assessment of Sebum levels [23].

Corbin and Marques [52] used ITA with four different approaches to estimate FST in three skin lesion image datasets: ISIC 2018, ISIC 2017, and ISIC 2016 [67], none of which provide information on skin types. Their study compares different methods for ITA‐based estimation and skin type classification, highlighting the impact of skin lesion size on their results. They proposed new methods based on AI models and ITA to address the lack of skin type information in skin image datasets lacking this metadata. Their research aims to address the underrepresentation of darker skin types in public skin lesion image datasets to produce fair models.

Kinyanjui et al. [31] used ITA to assign FST categories in other dermatology datasets that do not include Fitzpatrick skin type information: SD‐198 [68] and ISIC‐2018 [67]. They highlighted the limitation of the underrepresentation of darker skin populations in publicly available skin disease datasets. The skin type classification results showed that the majority of images belonged to lighter skin types. Campiche et al. [33]. used ITA and IWA_Newtone_ [69] to show the significant difference in skin type between these two groups of lighter‐ and darker‐skinned women. These two methods were compared in terms of skin color and signs of aging using the ColorFace imaging system. IWA_Newtone_ was developed by Newtone Technologies and incorporates components of luminance and chrominance in its assessment. There was a lack of some skin types, including lighter skin types and darker skin types.

As it was highlighted in the study by Alipour et al. [70]., the availability of large and diverse datasets is a major challenge in skin type research. There are two publicly available FST‐labeled datasets (e.g., PAD‐UFES‐20 and Fitzpatrick17k) that exhibit significant class imbalance and potential bias; using them to train deep learning models may reproduce and reinforce these biases. As a result, applying such models to these datasets may unintentionally propagate biased labeling and worsen the fairness issues the field aims to address. However, dermatologist‐assigned FST labels derived from the PAD‐UFES‐20 dataset were used in our study, providing a more consistent basis for evaluation.

According to the explanations above regarding skin type measurement methods, most existing literature either overlooks the influence of varying lighting conditions on their results or fails to assess their methodologies across all six skin types. Therefore, it is necessary to assess the performance of skin type measurement methods in these challenging scenarios to understand how they respond to such conditions and identify their weaknesses. Among these methods, ITA has emerged as a widely used image‐derived color/lightness metric commonly mapped to Fitzpatrick skin type categories, particularly in studies involving image datasets without expert‐assigned skin type labels. However, its reliability under uncontrolled lighting conditions and imbalanced data remains insufficiently understood. Our study does not introduce a new measurement method but rather investigates ITA's limitations in accurately measuring skin type diversity and offers new insights into its practical performance. Unlike previous studies that addressed general biases and proposed broad solutions in skin type measurement methods, our work delves into the limitations of this method itself on an image dataset with Fitzpatrick labels. It is investigated whether ITA can reliably characterize dataset‐level skin color variation under uncontrolled imaging conditions. Due to the limited availability of datasets with expert‐assigned Fitzpatrick skin type labels, our study focused on PAD‐UFES‐20, which also offers clear separation between lesions and surrounding healthy skin, facilitating reliable skin patch extraction. This level of detailed analysis has not been a focus of previous research.

The major contributions in this work include:

1. A systematic review of skin type measurement methods, highlighting the widespread use of ITA as a proxy for image‐derived skin color in prior research.
2. Construction of a skin patch image dataset with dermatologist‐assigned Fitzpatrick skin type (FST) labels, enabling evaluation under real‐world imaging conditions.
3. Analysis of color and lightness characteristics across all six FST categories, providing insight into skin tone variation in image datasets.
4. A critical evaluation of ITA under lighting variability and dataset imbalance, to examine its practical behavior in real‐world scenarios.
5. Empirical comparison between ITA‐derived predictions and dermatologist‐assigned FST labels to assess their level of agreement.
6. Critical analysis of ITA's limitations for dataset‐level skin color characterization.

## 2 Method

**FIGURE 2:**
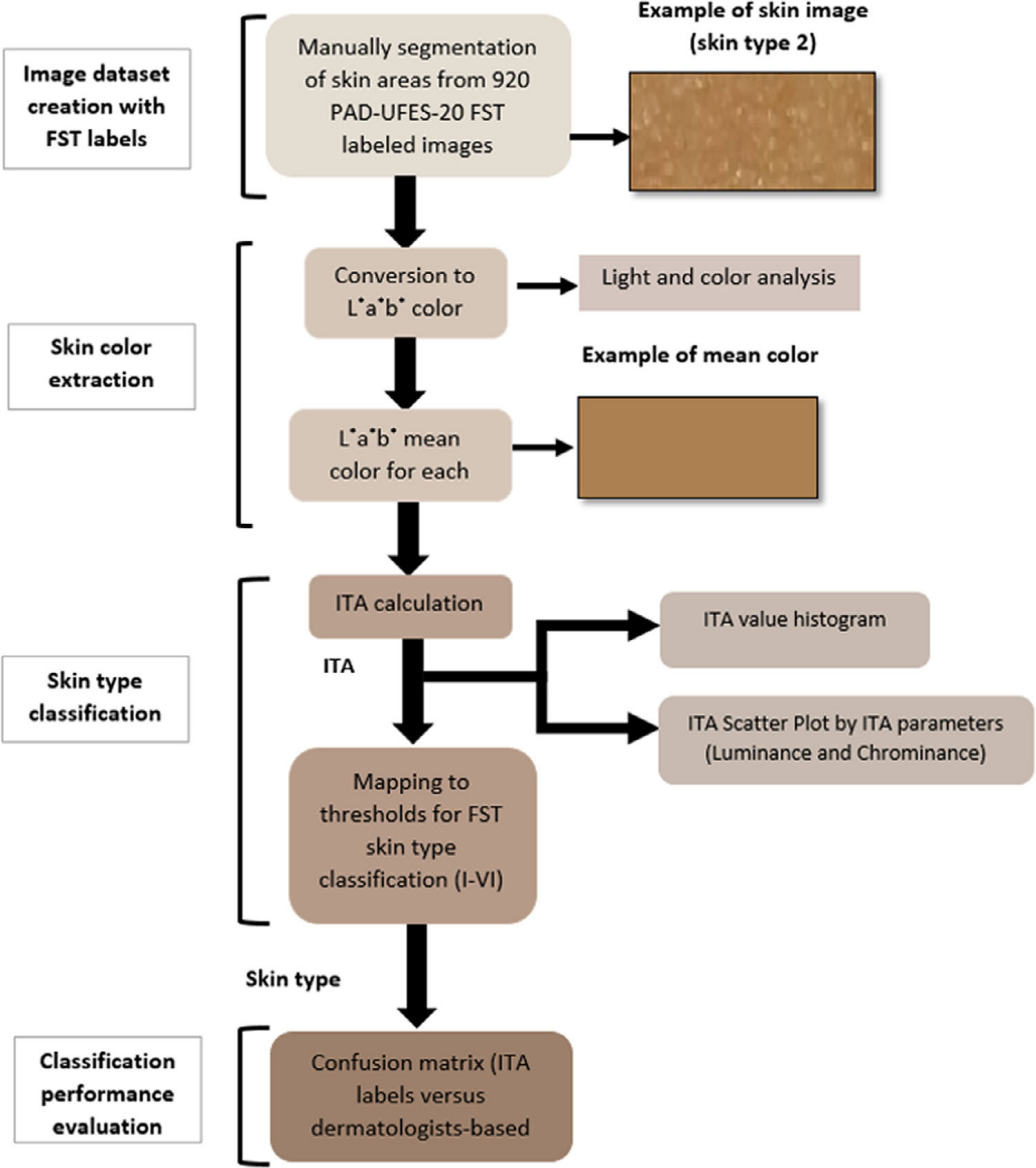
Flowchart of skin type measurement assessment. A dataset of 920 images is created, sourced from PAD‐UFES‐20, and labeled with FST. Skin patches are segmented and converted to L^*^a^*^b^*^ color space, and mean color values are extracted. ITA is calculated, mapped to thresholds, and classified into FST categories (I‐VI). Performance is evaluated using a confusion matrix based on FST ground truth labels.

The performance of ITA for the skin patch image dataset is evaluated using the method shown in Figure 2. This evaluation includes analysis of the distribution of ITA values across the dataset and within all six FST categories, as well as a comparison between ITA‐based predictions and dermatologist‐assigned FST labels. Although ITA is not inherently a skin type classifier, it is analyzed in a classification‐like setting to examine its behavior when mapped to Fitzpatrick categories. The dataset's color and lightness distributions were analyzed to gain insights into the variations of color and lightness across different skin types in the images. The approach involves several key steps, including dataset preparation, color space conversion, and ITA‐based FST classifications.

### 2.1 Image Dataset Creation

**FIGURE 3:**
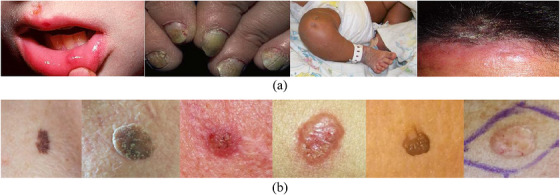
Some samples of images from (a) Fitzpatrick17k [72] and (b) PAD‐UFES‐20 [71].

The first step of the evaluation process is the creation of a dataset of healthy skin patch images, labeled with FST. To achieve this, existing skin lesion image datasets were reviewed to identify suitable candidates based on the following criteria: availability of FST labels, representation of all FST categories, and clear differentiation between skin and background. Two publicly available datasets, PAD‐UFES‐20 [71] and Fitzpatrick17k [72], were identified as potential sources and are shown in Figure 3(a)) and Figure 3(b)), respectively. PAD‐UFES‐20 was ultimately selected due to its distinct advantage over Fitzpatrick17k, as shown in Figure 3. Most images in PAD‐UFES‐20 display a clear separation between skin lesions and the background, facilitating reliable segmentation and extraction of healthy skin patches for the experiments. This dataset was used to manually generate skin patch images of different sizes for each FST category.

**FIGURE 4:**
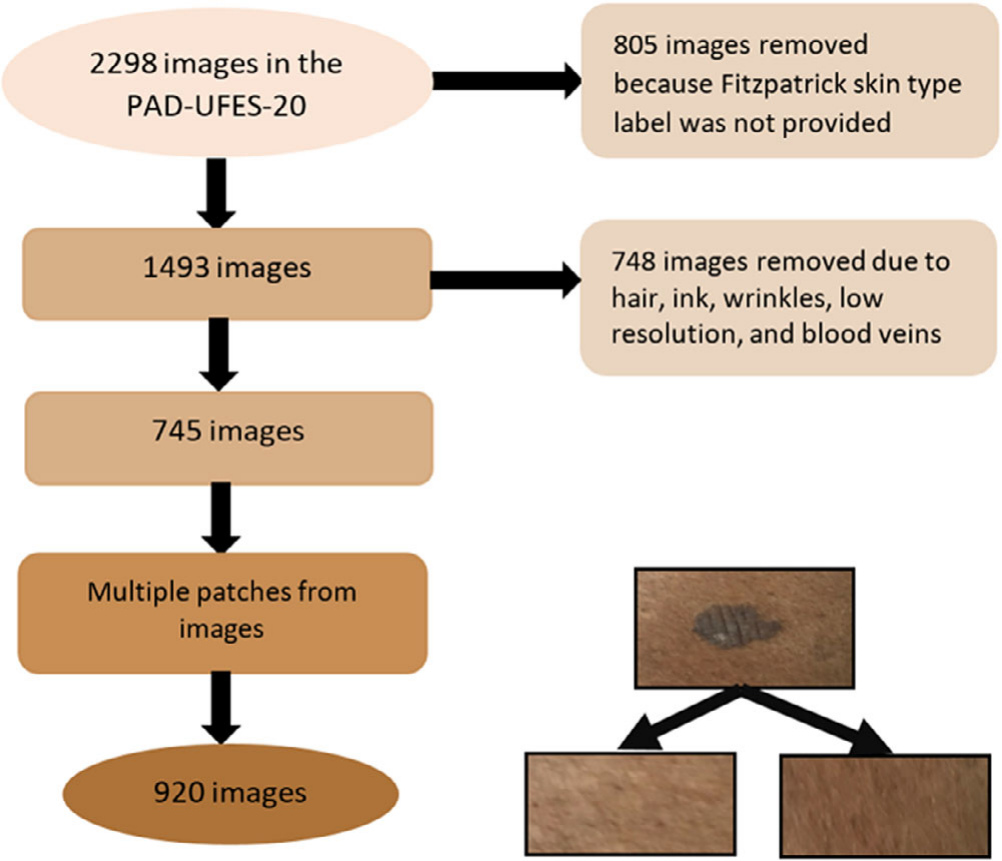
The process to create the skin patch image dataset for evaluating ITA as an image‐derived color/lightness measurement approach. Starting with 2298 images from the PAD‐UFES‐20 dataset, this process involves filtering images based on FST labels and visual quality, followed by manual extraction of skin patches.

It should be noted that both datasets are imbalanced in favor of lighter skin types and have images of different sizes. In the process of generating skin patch datasets from PAD‐UFES‐20, a systematic approach was followed, as shown in Figure 4.

The PAD‐UFES‐20 dataset contains 2298 images of skin lesions of varying sizes, originally collected for the diagnosis of six different skin conditions. However, this study focuses only on the healthy skin surrounding the lesions to create a dataset of skin patches labeled with FST. Images containing significant amounts of hair, ink, or wrinkles that obscured the skin region of interest were excluded from consideration. However, images with minimal hair that did not interfere with skin visibility were retained to avoid unnecessary exclusion and potential bias. From each image, multiple skin patches were extracted based on avoiding regions with hair, ink, wrinkles, or other obstructions; ensuring representation of diverse lighting conditions when present; selecting areas with uniform texture to exclude scars or lesions; and maintaining consistent resolution across the dataset. These criteria ensured that the extracted patches represented healthy skin regions suitable for ITA‐based skin type classifier evaluation, as illustrated in Figure 3. Consequently, the resulting skin patch image dataset encompasses samples of varying sizes. This process aimed at creating six different skin patch image datasets, each corresponding to a specific FST. In the end, 920 images remained for the experiments. The distribution of each skin type for the created skin patch image dataset and the PAD‐UFES‐20 are shown in Table 2.

**TABLE 2:** Number of images in each skin type group (FST) within the PAD‐UFES‐20 and the created skin patch image dataset.

Dataset	Total number	Skin type I	Skin type II	Skin type III	Skin type IV	Skin type V	Skin type VI
PAD‐UFES‐20	2298	153	876	392	62	10	1
Skin patch dataset	920	110	472	277	46	12	3

**FIGURE 5:**
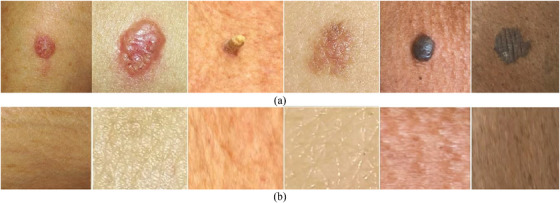
(a) Six samples of skin lesion images from Pad‐UFES‐20, (b) their corresponding skin patch images. (left to right) Skin types (FST) I‐VI, respectively.

Figure 5(a)) presents sample original images from the PAD‐UFES‐20 dataset, while Figure 5(b)) displays the corresponding skin patch images extracted from these samples, representing different skin types in the skin patch image dataset. The images in the PAD‐UFES‐20 present a wide range of lighting conditions, including shadowed areas, uneven lighting, and local overexposure.

### 2.2 Skin Color Extraction

Convert to L^*^a^*^b^*^ color space: The skin patch images were converted from RGB into the L^*^a^*^b^*^ color space, a color space defined by the International Commission on Illumination (CIE) in 1976 [73]. Luminance (L^*^) represents the lightness of the color, while chrominance components (a^*^ and b^*^) capture the color variations along the green‐red (a^*^) and blue‐yellow (b^*^) axes, independent of lightness [73]. This color space is designed to be more consistent with human vision and enhances the precision of measurements by reducing the impact of lighting conditions, a common issue in the RGB color space. The color space conversion is performed on each pixel in each skin patch image. The first step involves converting the RGB values (from 0 to 255) to XYZ using a linear transformation [73, 74] as follows:

(1) $$\left(\begin{array}{c} X\\ Y\\ Z \end{array}\right)=\left(\begin{array}{ccc} 0.4124 & 0.3576 & 0.1805\\ 0.2126 & 0.7152 & 0.0722\\ 0.0193 & 0.1192 & 0.9505 \end{array}\right)\left(\begin{array}{c} R\\ G\\ B \end{array}\right)$$

The L^*^a^*^b^*^ color space components are calculated from the XYZ using the following equations:

(2) $$L^{*}=\left\{\begin{array}{c} 116.{\left(\frac{Y}{Y_{n}}\right)}^{\frac{1}{3}}-16,if\;\frac{Y}{Y_{n}}>0.008856\\ 903.3\left(\frac{Y}{Y_{n}}\right),if\;\frac{Y}{Y_{n}}\leq 0.008856 \end{array}\right\}$$

(3) $$a^{*}=500\left[{\left(\frac{X}{X_{n}}\right)}^{\frac{1}{3}}-{\left(\frac{Y}{Y_{n}}\right)}^{\frac{1}{3}}\right]$$

(4) $$b^{*}=200\left[{\left(\frac{Y}{Y_{n}}\right)}^{\frac{1}{3}}-{\left(\frac{Z}{Z_{n}}\right)}^{\frac{1}{3}}\right]$$

Where $X_{n},Y_{n},Z_{n}\;$ represent the tristimulus values associated with the illuminant, which the human eye perceives as “white” [75]. The maximum value of L^*^ is 100 (white) and the minimum is 0 (black). The b^*^ axis indicates yellow to blue, and a^*^ axis shows red to green, with values ranging from ‐128 to +127 for each.

**FIGURE 6:**
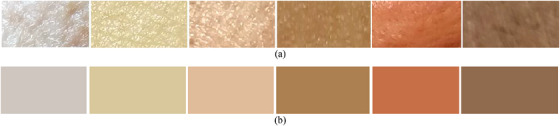
From left to right: (a) images from skin types (FST) I to VI, respectively. (b) Each image shows the corresponding estimated skin tone.

Image mean L^*^a^*^b^*^ value: The L^*^a^*^b^*^ mean value represents the average values of the L^*^, a^*^, and b^*^ channels across all pixels in each image within the skin patch dataset. This provides a consistent color representation for each image by averaging out local variations or anomalies, such as noise, lighting inconsistencies, or minor irregularities in the image. Although the mean values are visualized as solid‐color images, they are fundamentally single‐color values calculated for each image. Figure 6(a)) shows examples of images from the skin patch dataset alongside their corresponding extracted skin color for six FSTs in Figure 6(b)).

*ITA calculation*: The ITA method provides a numerical representation of skin color using the key colorimetric parameters L^*^ and b^*^ in the L^*^a^*^b^*^ color space. This method has emerged as a widely used image‐derived color metric commonly mapped to Fitzpatrick categories [10]. To quantify skin color, ITA measure is calculated from the mean Lab images as shown in equation (5):

(5) $$ITA=\tan^{-1}\left(\frac{L^{*}-50}{b^{*}}\right)\times \frac{180^{0}}{\pi }$$

**FIGURE 7:**
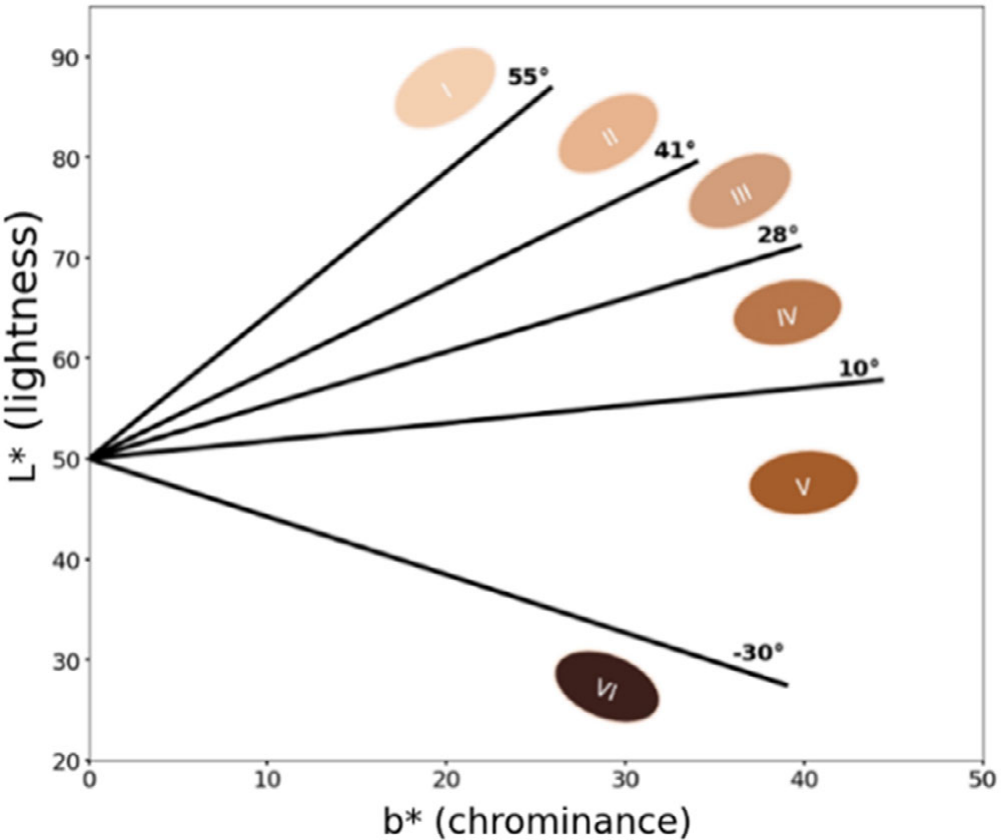
Partitioning of the ITA spectrum into six discrete categories (FST I‐VI) based on predefined thresholds [76], which reflect variations in lightness (L^*^) and chrominance (b^*^).

This method was originally proposed by Chardon et al. [76] to categorize different skin types based on ITA values. Higher ITA values indicate lighter skin types. The FST classification is determined by comparing the ITA values with predefined thresholds corresponding to specific skin type categories, as shown in Figure 7.

### 2.3 Skin Type Classification

*Mapping to thresholds*: ITA values were calculated from the previous step and were mapped to the predefined thresholds [76] to classify images into one of six FSTs according to Table 3. The thresholds were established using measurements from individuals with lighter skin types.

**TABLE 3:** Skin type (FST) classification based on ITA threshold values [76].

ITA range [°]	Predicted skin type
ITA>55°	Skin type I
41°< ITA ≤55°	Skin type II
28°< ITA ≤41°	Skin type III
10°< ITA ≤28°	Skin type IV
−30°< ITA ≤10°	Skin type V
ITA←30°	Skin type VI

## 3 Results

The experiments were conducted using Python 3.11.0 and various libraries such as OpenCV and Matplotlib. The skin patch image dataset was outlined in PNG format with image dimensions ranging from 59×67 to 2929×593. In section 3.1, the analysis of images in terms of color and lightness is assessed. In Section 3.2, the overall distribution of ITA values is analyzed to highlight skin color imbalances in the dataset. Section 3.3 examines ITA distributions within each Fitzpatrick skin type (FST) category. Section 3.4 explores the influence of ITA parameters (L^*^, b^*^) on their values. Section 3.5 compares ITA predictions with expert‐assigned FST labels using a confusion matrix. Finally, Section 3.6 presents examples of misclassified images and analyzes how lighting variation impacts ITA‐based classification accuracy. The code and dataset can be found here: https://github.com/NedaAlip/Assessment‐of‐skin‐type‐measurement‐method/tree/main

### 3.1 Color and Lightness Analysis of Skin Patch Images

**FIGURE 8:**
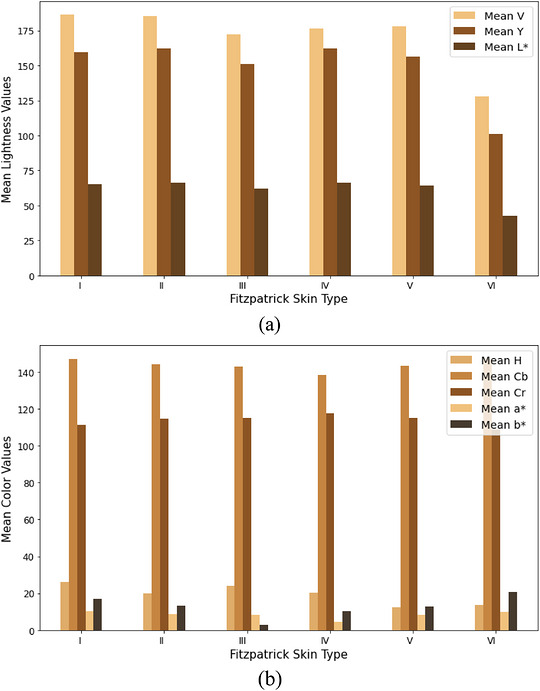
(a)‐(b). Comparison of mean lightness and color values in three color spaces of HSV, YCbCr, and L^*^a^*^b^*^. Each bar represents the mean value of the corresponding parameters for dermatologist‐assigned Fitzpatrick skin types (FST). Figures show that lightness values (V, Y, L^*^) exhibit some variation across FSTs, and a decrease in skin type VI. The color components (Cb, Cr) remain relatively stable, but H, a^*^, and b^*^ show changes over various skin types.

Figures 8 (a)‐(b) compare the lightness and color values in three color spaces across all skin types. The aim is to observe how lightness and color vary across different skin types and whether these parameters accurately suit measuring skin color diversity. Labels of type I to type VI refer to the different FSTs. These figures are shown in three HSV, YCbCr, and L^*^a^*^b^*^ color spaces. V, Y, and L^*^ parameters correspond to lightness, and H, Cb, Cr, a^*^, and b^*^ refer to the chrominance parameters.

Figures 8(a)) and 8(b) show that lightness decreases in all color spaces in type VI, reflecting the darker nature of these skin colors. The Hue values gradually decrease as the skin becomes darker. The Cb and Cr values show fewer variations across different skin types, whereas H, a^*^, and b^*^ values show more noticeable changes. In particular, H tends to decrease from type I to type VI, and b^*^ reaches its minimum value at type III.

**FIGURE 9:**
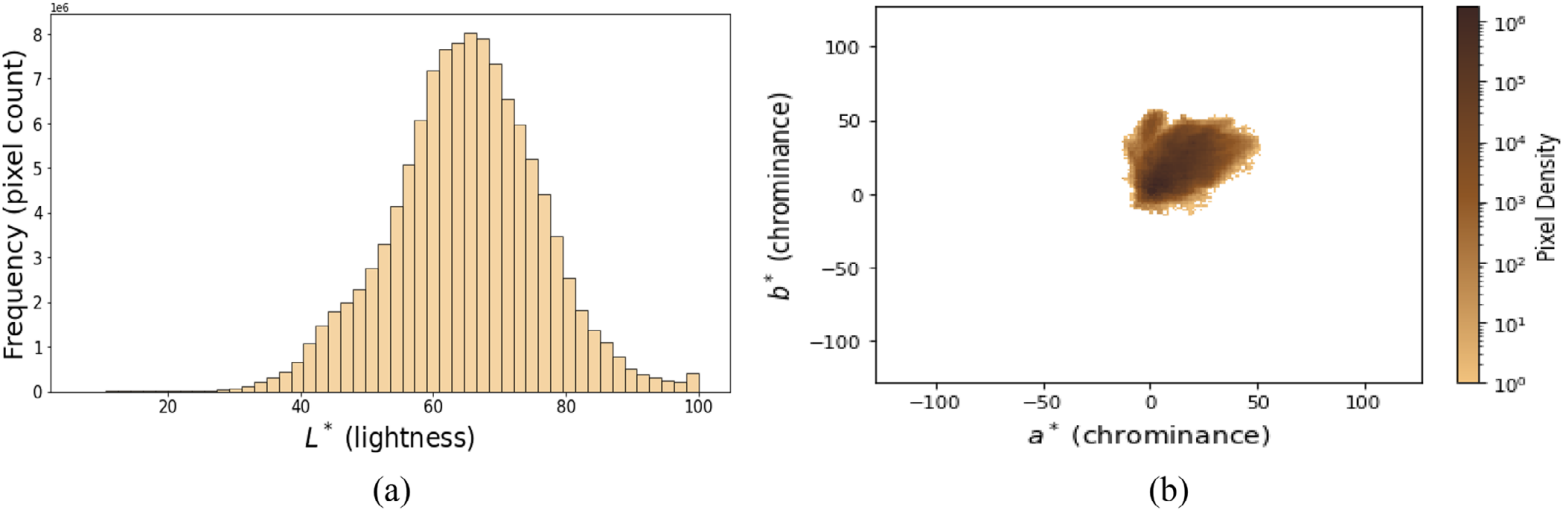
Pixel‐level‐based histogram showing the distribution of (a) lightness (L^*^) and (b) chrominance (a^*^ and b^*^) across all skin types in the created dataset.

Figure 9(a)) shows the lightness distribution across all skin types in the dataset, based on pixel counts. The lightness distribution in the range 60 ≤ L^*^ ≤ 80 appears symmetrical, suggesting that most pixels represent moderate L^*^ values.

The distribution suggests that most of the images in the dataset have relatively balanced lightness, neither too dark nor too light. In ranges L^*^<40 and L^*^>80, there are fewer pixels in very low lightness (very dark), and similarly, the number of pixels with very high lightness (very light) is also low. In Figure 9(b)), the 2D histogram illustrates the chrominance distribution of the dataset. Most pixels are concentrated near the center, indicating that most skin tones in the dataset have similar chromatic components, with chrominance values clustering closely together. The majority of them fall within the range of 0 to 50.

### 3.2 ITA Values Distribution across an Imbalanced Dataset

**FIGURE 10:**
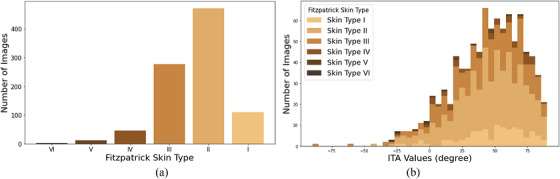
(a) Distribution of dermatologist‐labeled FSTs that show an imbalance in the skin patch dataset. (b) Histogram of ITA values of the skin patch image dataset indicating disparity in the estimated skin types across the dataset.

The effectiveness of ITA as a means of classifying skin patch images into FSTs was evaluated. The experiment assessed how ITA performs when faced with an imbalanced dataset with unequal representation of different FSTs. As the PAD‐UFES‐20 dataset is imbalanced, the skin patch dataset created from it was also imbalanced. Figure 10(a)) shows the skin patch image distribution in the dataset, and Figure 10(b)) shows the histogram of ITA values within that dataset. Figure 10(a)) is a visual representation that depicts an alarming under‐representation of darker skin types, and Figure 10(b)) reflects this deficiency with the near absence of data points in the negative ITA range values.

The histogram is left‐skewed, and the increasing density towards the peak of the histogram indicates that these ITA values are much more common in the dataset. The prominent peak occurs in the range of values between 35 and 65, corresponding to lighter skin types. This indicates that the dataset contains mostly ITA values associated with these two skin types. There is an initial decrease in frequency as ITA values move from the positive range towards the center. This decrease suggests that there are fewer images with darker skin types compared to lighter skin types. The bins corresponding to lower ITA values, representing darker skin types, are notably emptier than lighter skin types. Figure 10(b)) reveals an overlap between the ITA distributions of different Fitzpatrick skin types, suggesting that ITA may struggle to reliably separate Fitzpatrick skin types.

### 3.3 Distribution of ITA Values across each FST Category

**FIGURE 11:**
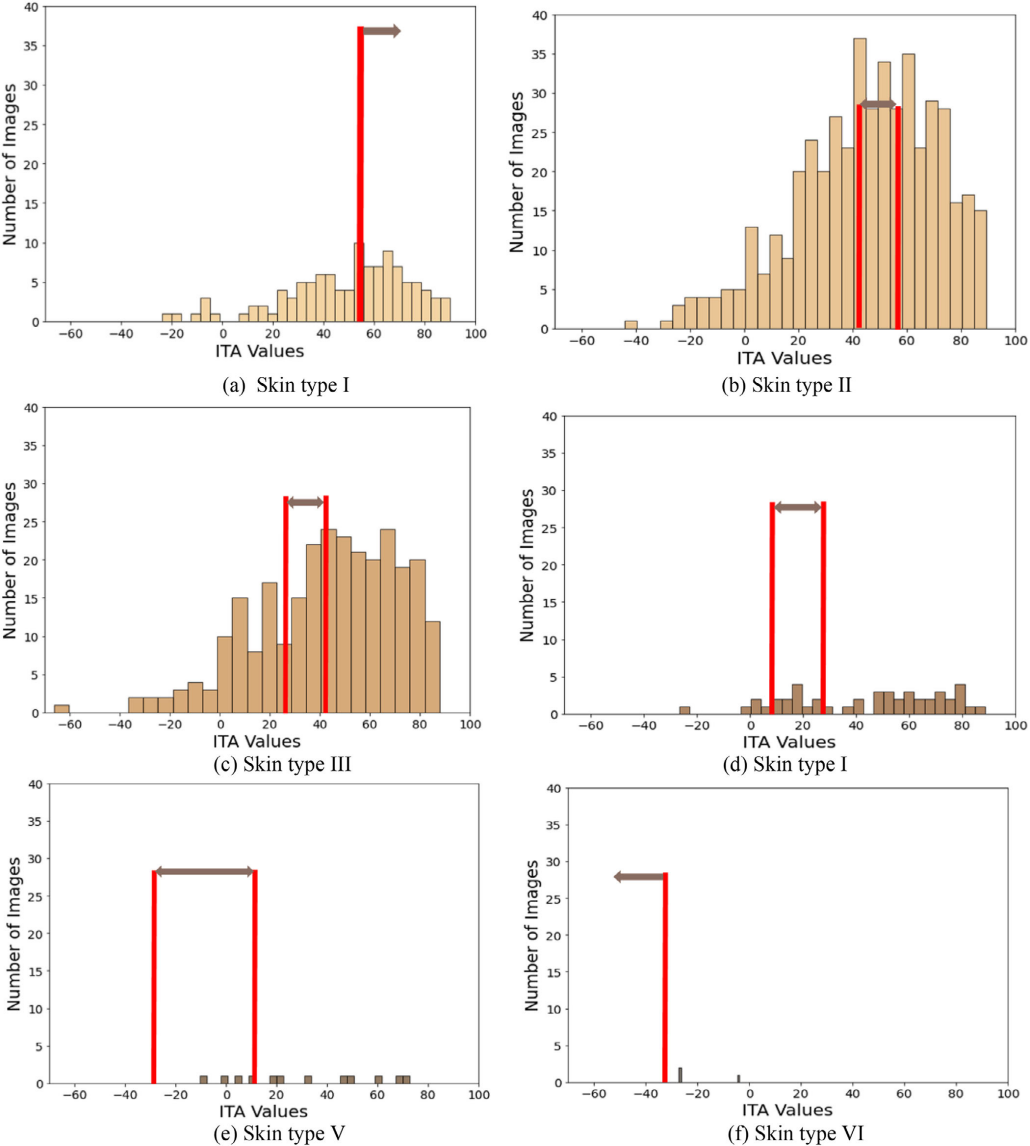
ITA histograms across each dermatologist‐based labeled skin type—(a)–(f): skin types I to VI, respectively, with predefined thresholds indicated by red lines. The blue arrows illustrate the range of expected ITA values for each skin type. The histograms reveal variations in ITA values and their deviations from the thresholds.

Concerning ITA's performance across different skin types, Figure 11(a)) to Figure 11(f)) present a series of histograms, each corresponding to a different skin type dataset, illustrating the distribution of ITA values. The red lines represent the Fitzpatrick skin type thresholds as defined by ITA values, based on [65] and presented in Table 3. The arrows indicate the range of ITA values corresponding to each skin type within these threshold domains.

Figure 11 shows that as the skin types darken, the distance between thresholds becomes greater, and also shows a decrease in the number of bins within threshold space, particularly in skin type VI, where there are no bins at the corresponding threshold domain. Therefore, ITA values related to each skin type do not fall exactly within the range related to that type of skin, indicating ITA values cannot follow the fixed thresholds.

### 3.4 Distribution of ITA Values vs L^*^ and b^*^ Parameters

**FIGURE 12:**
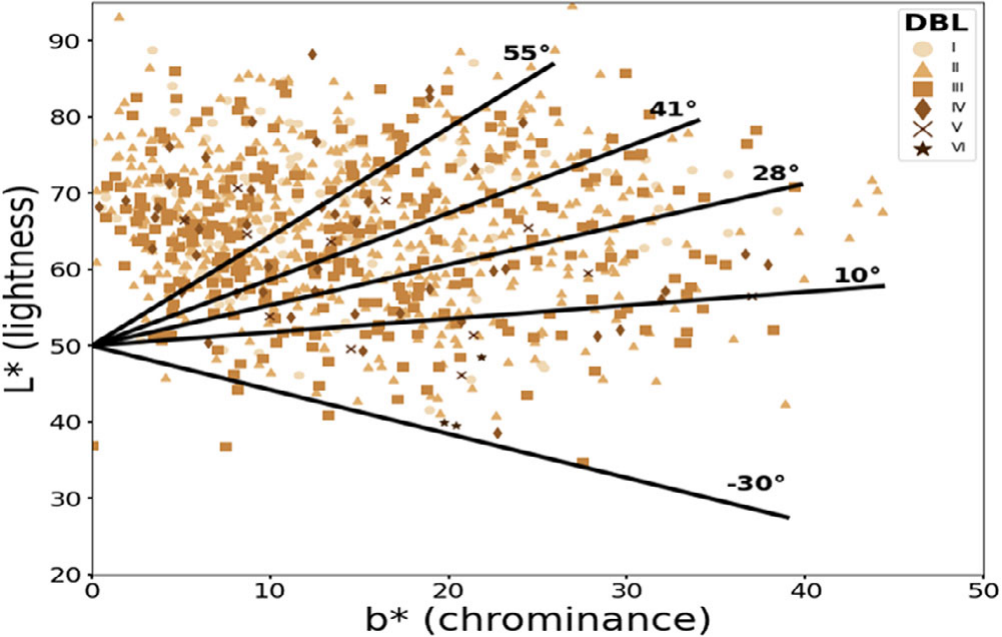
Scatter plot of ITA values according to the dermatologist‐based labels (DBL) versus the mean of L^*^ and b^*^ parameters. The background shows six different divided sectors, each corresponding to a range of ITA values. It shows how the ITA‐based classifier assigns images to skin type classes based on the ITA value ranges (rays). Points within the same ray are classified into the same skin type category.

Figure 12 shows the assessment of ITA values' dependence on luminance and chrominance parameters over different skin types, each distinguished by a unique shape.

In Figure 12, the densest concentration of data points appears to be in the upper areas of the figure with higher luminance range and moderate chrominance values, which could indicate that most of the measured skin types belong to lighter skin types. At the same time, the distribution of points shows the overlap between different skin types, particularly lighter skin types, showing a challenge for the method to effectively distinguish between them and increasing the rate of misclassification for lighter skin types. For skin type VI, it shows no correct classification. The results indicate that ITA‐based classification in this way is not suitable for reliably assigning images to different FSTs. The classifier assigns images based on predefined thresholds (rays), but these assignments do not correspond accurately to the actual skin types as determined by dermatologist‐assigned ground truth labels.

### 3.5 ITA Prediction vs Dermatologist‐assigned Labels

**FIGURE 13:**
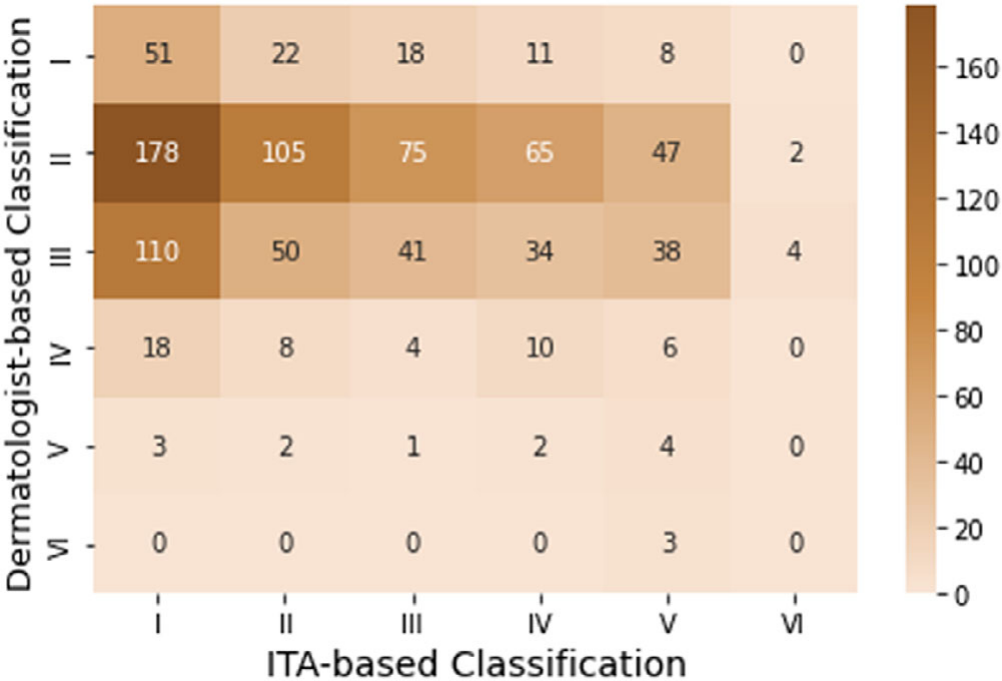
Confusion matrix using the skin patch image dataset. Each cell represents the number of samples from the predicted skin type using ITA method (columns) against the Fitzpatrick dermatologist‐based skin type (rows). The matrix illustrates the method's performance, highlighting the accuracy and misclassification rates for different skin types.

Another method for evaluating the performance of an ITA‐based classifier is by using a confusion matrix, which compares its predictions against dermatologist‐assigned FST labels (ground truth), which is shown in Figure 13. This matrix compares the results of the ITA‐based skin type classification versus a dermatologist‐based one across six FSTs. Each column shows results for ITA, while rows show results for dermatologist‐based classification, and the diagonal elements from the top left to bottom right indicate the number of occurrences where both systems have the same results for classification.

The confusion matrix highlights substantial misclassification across all Fitzpatrick skin types, with fixed‐threshold ITA showing very limited capability to distinguish between dermatologist‐assigned FST categories. Lighter skin types, particularly type II, were frequently misclassified as neighboring categories such as type I, while the darkest skin type (FST VI) received no correct classifications. Overall, fixed‐threshold ITA achieved 22.9% classification accuracy, which is below the majority‐class baseline accuracy of 51.3%. These findings demonstrate that fixed‐threshold ITA does not provide meaningful discriminatory performance for Fitzpatrick skin type classification under uncontrolled imaging conditions.

### 3.6 Statistical Analysis of ITA Values across Skin Types

To support the statistical analysis of ITA performance across skin types, the mean ITA and the corresponding 95% confidence interval (CI) for each dermatologist‐assigned Fitzpatrick group were calculated as shown in Table 4. The CI provides an estimate of the uncertainty around the mean ITA: the lower bound (CI Low) and upper bound (CI High) delimit the range in which the true mean ITA value is expected to lie with 95% confidence.

**TABLE 4:** Mean ITA values and 95% confidence intervals across dermatologist‐assigned FST labels.

Skin type (FST)	Mean ITA	Number of images	95% CI (Low)	95% CI (High)
I	49.04	110	44.43	53.66
II	44.90	472	42.58	47.22
III	42.85	277	39.42	46.27
IV	43.87	46	35.60	52.15
V	30.89	12	15.01	46.77
VI	−19.24	3	−34.36	−4.12

As shown in Table 4, types V and VI exhibit considerably wider intervals due to the small number of available samples, indicating reduced reliability of ITA estimates for darker skin types.

### 3.7 Examples of Misclassified Images

**FIGURE 14:**
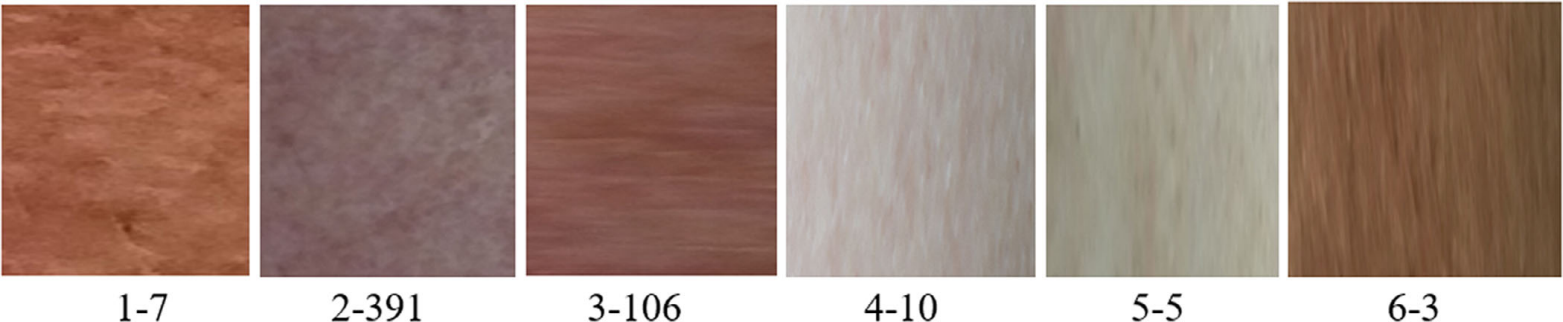
Examples of misclassified images due to varying lighting conditions. They are listed along with their corresponding numbers within the skin patch dataset.

Figure 14 and Table 5 illustrate sample images that were misclassified by the ITA method. These examples show how lighting variations, such as uneven lighting, reflections, or shadows, can alter perceived skin color and lead to incorrect ITA calculations.

**TABLE 5:** Summary of misclassification causes, ITA values, and discrepancies between ITA‐based predictions and dermatologist‐assigned skin type labels.

Cause of misclassification	ITA value	Prediction by ITA	Dermatologist‐based label	Image number
Low light/shadow	−6.561	5	1	1–7
Uneven lighting, reddish tint	−44.238	6	2	2–391
Side lighting/reflection loss	−34.352	6	3	3–106
Overexposure/strong light	78.586	1	4	4–10
Bright ambient light/flash	59.302	1	5	5–5
Slight overexposure or reflection effect	−27.107	5	6	6–3

This table presents examples of misclassified images to analyze the reasons behind ITA errors. The results indicate that lighting conditions such as low light, reflection, uneven lighting, and direct exposure altered ITA values, leading to incorrect skin type classification.

## 4 Discussion

Our study focused on evaluating ITA's robustness and reliability as a continuous quantitative metric of skin color analysis in image datasets. Given the limited access to datasets with expert‐labeled Fitzpatrick types, PAD‐UFES‐20 was chosen for its well‐separated lesion and healthy skin areas, allowing for better skin patch segmentation. The number of images labeled as skin types V or VI in the PAD‐UFES‐20 dataset (and hence in our skin patch image dataset) is very low. The distribution of ITA values across lighter skin types (higher ITA values) and darker skin types (lower ITA values) reflects the over‐representation of light skin types and under‐representation of dark skin types. Also, the skin images in PAD‐UFES‐20 present a wide range of lighting conditions, including shadowed areas, uneven lighting, and local overexposure. The results indicated that while the ITA method provides measurable differences in lightness across the dataset, its ability to assign FST categories accurately based on FST was limited not only by dataset imbalance, but also by substantial overlap between ITA distributions, predictions misalignment, lighting sensitivity, and failure to correctly classify the darkest skin type (FST VI).

**FIGURE 15:**
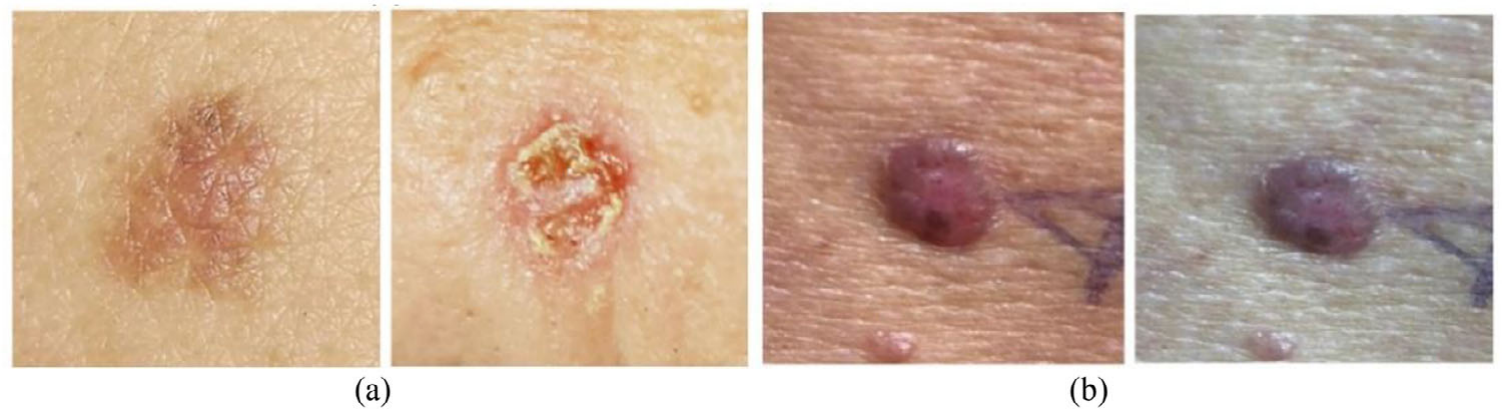
Four image samples from PAD‐UFES‐20 show a misperception of skin color due to lighting variations. (a) Two images from different skin types (IV and III) show a perceived similarity in skin color due to lighting conditions. (b) Two images of the same patch of skin (I), taken under different lighting conditions, demonstrate significant variability in perceived skin color.

**FIGURE 16:**
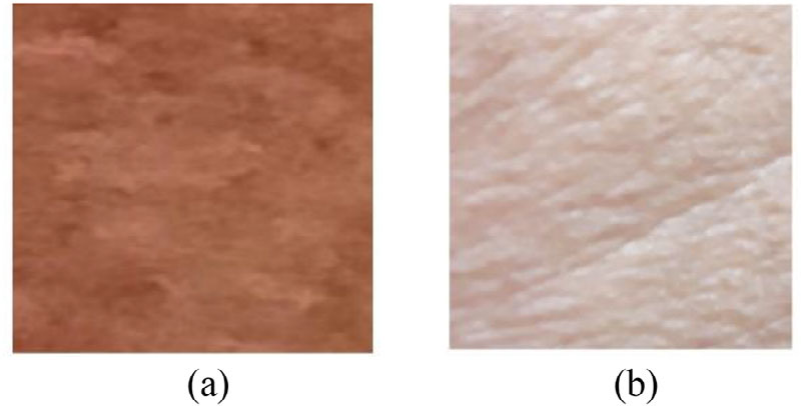
Two samples from the skin patch image dataset show how lighting conditions affect how skin colors appear. (a) The image of skin type I looks like dark skin type. (b) The image is from skin type V, which looks like a lighter skin type.

The histogram distribution of ITA values for each FST showed that ITA does not conform well to the predefined FST ranges, particularly for darker types. The broader range of ITA values compared to the FST thresholds suggests that ITA produces broader continuous variation than discrete FST thresholds. Lighting variations further exacerbate ITA‐based classifier's limitations, as shown in Figure 15 and Figure 16, where the same skin type appears differently under different lighting conditions.

Figure 15(a)) illustrates that under varying lighting conditions; different lighter skin types may exhibit similar reflection and absorption properties. This results in more overlapping values within ITA method's parameter space, leading to misperceptions of true skin color and confusion with other light skin types. The higher rate of misclassifications between adjacent lighter skin types I and II in the confusion matrix highlights this issue. Figure 15(b)) illustrates why ITA‐based method classifies the same skin types across different thresholds, instead of classifying them within the same threshold range. It suggests that ITA‐based classifier is primarily influenced by changes in lightness (L^*^), although it is also affected by variations in chrominance (b^*^), depending on the extent of those variations. Figure 16(a)) and Figure 16(b)) illustrate how lighting variations can alter the perceived skin color, leading to misclassification of skin types. Accurate lighting calibration and standardized imaging conditions are essential to minimize such inconsistencies and improve the reliability of skin type measurement methods.

Figures 15 and 16 demonstrated how lighting conditions, such as overexposure, shadowing, and uneven lighting conditions, alter the perceived color and lightness of skin, leading to misclassification. Lighter skin types (I and II), which reflect more diffuse light, exhibited relatively stable ITA values under variable lighting. In contrast, darker skin types, which absorb more light due to higher melanin concentration, showed greater variability in ITA measurements, particularly in the L^*^ channel of the L^*^a^*^b^*^ color space [77]. This confirms that ITA is disproportionately affected by lighting when evaluating darker skin types.

The results also indicate that lighting variations disproportionately affect ITA measurements for darker skin types (V‐VI). Because ITA is driven by the L^*^ component of the L^*^a^*^b^*^ color space, any reduction in lightness caused by shadows, uneven lighting, or optical absorption is amplified in darker skin, where L^*^ values are already low. As a result, small lighting changes can cause large shifts in ITA values, making the method less stable for highly pigmented skin. This instability is clearly visible in Figure 11 (e–f), where the ITA histograms for types V‐VI show wide dispersion and poor alignment with the expected threshold ranges. These findings highlight that ITA mapping is inherently less robust for darker skin because reduced reflectance and higher melanin absorption magnify lighting‐dependent fluctuations in L^*^. Results show that ITA is more sensitive to lighting variability in darker skin types, which limits its reliability when used in uncontrolled imaging environments.

The lightness histogram exhibits a broader distribution, reflecting variability in lighting conditions and highlighting ITA's greater sensitivity to lightness over chrominance. The low number of data points in the bins for very dark and very light values suggests the need for more samples to improve the analysis of these skin types. Table 2 indicates a clear dataset imbalance, with very few samples for skin types V and VI. This limits the strength of the conclusions that can be drawn for darker skin types and reduces the reliability of distributional estimates for these groups. However, the unstable ITA distributions observed for darker skin types should be interpreted as the combined result of small sample size, lighting sensitivity, overlapping ITA distributions, and mismatch between fixed ITA thresholds and dermatologist‐assigned FST labels, rather than as a consequence of dataset imbalance alone. As shown in Table 4, skin types V and VI exhibit wider 95% confidence intervals compared to lighter skin types. This reflects the combined effect of small sample sizes and greater lighting sensitivity in darker skin types. The broad intervals indicate that ITA‐based predictions for darker skins are less stable and less reliable. Also, overlapping ITA values across different FSTs in the histogram showed that FST skin type classification based only on ITA is not reliable.

Lightness also decreases in the darkest skin type of VI, while chrominance values show little variation, indicating limited color diversity and nuanced differences in representation between different skin types in the dataset. This lack of color diversity reflects the inherent similarity in chrominance values across all skin types, rather than the dominance of any specific skin type in the dataset.

ITA values were compared against Fitzpatrick labels assigned by dermatologists, which are discrete categories based on in‐person assessment. ITA showed very low classification accuracy when evaluated against subjective and discrete dermatologist‐assigned FST labels, which were used as the ground truth for the analysis. It does not accurately classify images into actual skin types as determined by dermatologist‐assigned labels. The inherent difference between these two continuous and discrete methods limits the direct alignment between ITA and FST. ITA's limitations in aligning with predefined FST thresholds highlight the need for alternative or enhanced metrics that are both robust to lighting changes and sensitive to subtle color variations, especially in darker skin colors. It shows that relying on fixed, discrete thresholds (such as those used in the Fitzpatrick system) may not be sufficient for identifying nuanced skin color variation. This limitation is particularly relevant for dataset fairness assessment, as underestimation or misclassification of darker skin types can cause bias in AI models. This highlights the need for alternative or complementary approaches capable of capturing the complexity of skin color diversity in image datasets.

Beyond ITA, other approaches have been proposed for skin type assessment. Physical and colorimetric methods, such as spectrophotometer‐based melanin or erythema indices, can provide accurate and lighting‐independent measurements; however, they are impractical for large‐scale image datasets due to their cost, acquisition time, and the need for controlled measurement conditions. More recent AI‐based methods may provide flexible and data‐driven approaches for skin color estimation; however, their performance also depends on the availability of sufficiently diverse and reliably labeled datasets, particularly for underrepresented darker skin types. These limitations help explain why ITA has been commonly used in prior work; however, its application should be treated with caution, particularly under varying lighting conditions, and only with explicit awareness of its inherent limitations, rather than as a standalone solution.

Threshold recalibration, lighting normalization, incorporating greater diversity, particularly within underrepresented skin colors, and preprocessing approaches may be explored in future work; however, the current results do not demonstrate that such modifications would be sufficient to achieve reliable Fitzpatrick skin type classification. PAD‐UFES‐20 was the only publicly available dataset that provided both dermatologist‐assigned Fitzpatrick labels and high‐resolution skin lesion images suitable for isolating skin regions. This issue restricted our ability to evaluate ITA across multiple datasets. It is considered a limitation, and it should be addressed in future work through collecting additional samples from darker skin types to ensure more balanced representation. Controlled lighting conditions and lighting normalization techniques would be valuable in future evaluations to minimize lighting‐related variability. Several preprocessing techniques have been proposed to address lighting variability in skin image analysis, including color constancy algorithms [78, 79], histogram equalization [80], and GAN‐based illumination normalization [81]. These methods may help reduce lighting artifacts and may improve the consistency of color‐based metrics such as ITA. In future work, integrating such preprocessing steps before ITA computation may allow for more robust and fair assessments.

Our comparison between ITA‐based predictions and dermatologist‐assigned FST labels revealed inconsistencies, largely due to dataset limitations such as class imbalance, rigid Fitzpatrick thresholds, and the sensitivity of ITA to lighting conditions. Although ITA provides a continuous representation of skin color, this is insufficient for reliable alignment with discrete dermatologist‐assigned FST labels. These results indicate that ITA is not suitable for reliable FST classification under real‐world imaging conditions. While ITA provides continuous image‐derived color representation, it cannot reliably characterize dataset‐level skin color diversity or fairness under uncontrolled imaging conditions. To fairly evaluate the performance of that, future work will focus on collecting a diverse dataset with controlled lighting conditions to support accurate evaluation. Such a dataset may support future investigation of threshold recalibration and evaluation of alternative continuous skin color metrics. Also, new ITA thresholds designed specifically for darker skin types should be explored, and ITA should be compared against emerging metrics and spectrophotometric ground‐truth measurements. Finally, using a calibrated imaging system would enable more reliable cross‐dataset comparisons and support the development of lighting‐aware skin tone assessment models. As part of future work, a new dataset has been designed as part of this work, with continuous skin color labels obtained using a colorimeter as a reference, along with controlled and well‐documented lighting conditions.

ITA has been used in dermatology and AI‐based skin analysis studies as an image‐derived skin color or lightness metric commonly mapped to Fitzpatrick categories [35, 50]. Recent studies also highlight the persistent under‐representation of darker skin in dermatology datasets and AI‐generated images [1, 82]. Our findings support these concerns, showing that reliance on ITA alone may introduce systematic bias, particularly when datasets lack adequate dark skin representation. The results further demonstrate that ITA is unreliable for FST classification under uncontrolled imaging conditions and should not be interpreted as evidence of reliable skin type assessment performance.

To assess skin type diversity in datasets used to train AI models and for capturing the detailed color gradations of different skin types, the Fitzpatrick labeling system, based on fixed and rigid thresholds, may not be sufficiently informative. Instead, there is a need to investigate more robust and validated approaches capable of capturing subtle variations in skin color and provide a more nuanced understanding of dataset diversity. The findings suggest that rigid categorical classifications alone may be insufficient for representing the full variability of skin color in image datasets. Given the importance of image‐derived skin color assessment methods in detecting any under‐representation within datasets, it is essential to ensure their performance under varying lighting conditions. This may help identify under‐representation before datasets are used to train AI models to ensure fair and unbiased performance of these models across all skin colors. The results indicate that fixed‐threshold ITA does not provide sufficiently reliable performance for FST classification under uncontrolled imaging conditions. It emphasizes the importance of developing and validating more robust approaches for image‐based skin color assessment, particularly under variable lighting conditions and across diverse skin colors.

## Funding

This publication has emanated from research supported by a grant from Science Foundation Ireland under Grant number 18/CRT/6222.

## Ethics Statement

This study did not involve any new data collection involving human participants. All datasets used (PAD‐UFES‐20 and Fitzpatrick17k) are publicly available. https://doi.org/10.13039/501100001602

## Conflicts of Interest

The authors declare no conflicts of interest.

## Data Availability

The data that support the findings of this study are openly available in Github at https://github.com/NedaAlip/Assessment‐of‐skin‐type‐measurement‐method/tree/main.
